# Supplementary material for: Non-linear bistability in pulsed optical traps
Source: Nanophotonics. 2025 Apr 24;14(23):4153–61. doi: 10.1515/nanoph-2025-0025 (PMC12617703; doi:10.1515/nanoph-2025-0025)
Supplement: Supplementary file 1 — Supplementary Material Details [file j_nanoph-2025-0025_suppl_001.pdf]

Supplementary information
Non-linear bistability in pulsed optical traps

1 Infinite cylinder illuminated with a plane wave

We consider plane wave illumination of an infinite cylinder for transverse electric (TE, the electric field vector is perpendicular to the plane of incidence) polarisation. In this case, the electric field vector lies in the same plane as the cylinder cross section. We are interested in the scattered and internal fields of the cylinder, which under normal incidence are two-dimensional (2D), i.e., uniform along the cylinder [1].

Assuming the cylinder axis is in the z direction and its circular cross section is in the xy plane, centred on $(x, y) = (0, 0)$, the scattered and internal fields can be expanded in cylindrical vector harmonics, denoted by $\mathbf{M}_n(\rho, \phi, z)$ and $\mathbf{N}_n(\rho, \phi, z)$ where $\rho = \sqrt{x^2 + y^2}$ and ϕ is the azimuth angle measured from the positive x axis (with the positive y axis at $\phi = \pi/2$), and are exact up to a chosen maximum order $n_{\max}$ that truncates the expansion. The direction of an incoming plane wave has two angles: θ is the angle of incidence with respect to z and φ is the plane wave azimuthal angle of incidence (note the distinction between this angle φ and the azimuth co-ordinate ϕ). The cylindrical vector harmonics relevant to a plane wave with a general incident wavevector $\mathbf{k} = -k(\sin\theta\cos\varphi\hat{\mathbf{x}} + \sin\theta\sin\varphi\hat{\mathbf{y}} + \cos\theta\hat{\mathbf{z}})$ are

$$\mathbf{M}_n(\rho, \phi, z) = nk\sin\theta\left(\mathrm{i}n\frac{Z_n(a)}{a}\hat{\boldsymbol{\rho}} - Z_n'(a)\hat{\boldsymbol{\phi}}\right)\mathrm{e}^{\mathrm{i}[n(\phi-\varphi)-k\cos\theta z]}, \tag{S1}$$

$$\mathbf{N}_n(\rho, \phi, z) = k\sin\theta\left(-\mathrm{i}Z_n'(a)\cos\theta\hat{\boldsymbol{\rho}} + n\frac{Z_n(a)}{a}\cos\theta\hat{\boldsymbol{\phi}} + Z_n(a)\sin\theta\hat{\mathbf{z}}\right)\mathrm{e}^{\mathrm{i}[n(\phi-\varphi)-k\cos\theta z]}. \tag{S2}$$

The function $Z_n(a)$ is a solution to Bessel's equation with argument a, to be specified later depending on the field being calculated, while $Z_n'(a)$ denotes the derivative with respect to a.

In the next three sections, we will derive the scattered and internal fields of the cylinder, $\mathbf{e}_{\mathrm{sca}}$ and $\mathbf{e}_{\mathrm{int}}$, respectively, under a unit-amplitude incident plane wave illumination $\mathbf{e}_{\mathrm{inc}}$ (hence the lower case $\mathbf{e}$ notation). The total field resulting from the interaction between a plane wave of amplitude A and the cylinder is

$$\mathbf{E}_{\mathrm{tot}}(\rho, \phi, z) = A\mathbf{e}_{\mathrm{tot}}(\rho, \phi, z) = \begin{cases} A[\mathbf{e}_{\mathrm{inc}}(\rho, \phi, z) + \mathbf{e}_{\mathrm{sca}}(\rho, \phi, z)] & \text{for } \rho > \rho_{\mathrm{cyl}} \\ A\mathbf{e}_{\mathrm{int}}(\rho, \phi, z) & \text{for } \rho \le \rho_{\mathrm{cyl}}\,, \end{cases} \tag{S3}$$

where ρ_{cyl} is the radius of the cylinder.

1.1 Incident field

Although the incident plane wave field is trivial to define, i.e.,

$$\mathbf{e}_{\mathrm{inc}} = (-\sin\varphi\hat{\mathbf{x}} + \cos\varphi\hat{\mathbf{y}})\mathrm{e}^{-\mathrm{i}k[\sin\theta\cos\varphi x + \sin\theta\sin\varphi y + \cos\theta z]}, \tag{S4}$$

it is useful to expand $\mathbf{e}_{\mathrm{inc}}$ in cylindrical vector harmonics to ensure the cylinder-free space boundary conditions are met for any given order n. The expansion of the incident field is given by

$$\mathbf{e}_{\mathrm{inc}}(\rho, \phi, z) = -\mathrm{i}\sum_{n=-\infty}^{\infty}\frac{(-\mathrm{i})^n}{k\sin\theta}\mathbf{M}_n(\rho, \phi, z)\,, \tag{S5}$$

where in this case the function $Z_n(a)$ in Eq. (S1) is the Bessel function of the first kind $J_n(a)$ and $a = k\rho\sin\theta$.

1.2 Scattered field

The scattered electric field is given by

$$\mathbf{e}_{\rm sca}(\rho,\phi,z) = \sum_{n=-\infty}^{\infty} \frac{(-{\rm i})^n}{k\sin\theta}\left[{\rm i}c_n\mathbf{M}_n(\rho,\phi,z) + d_n\mathbf{N}_n(\rho,\phi,z)\right] , \tag{S6}$$

where the function $Z_n(a)$ in the both Eqs. (S1) and (S2) is the Hankel function of the first kind, $H_n(a) = J_n(a) + {\rm i}Y_n(a)$, and the argument $a = k\rho\sin\theta$. The coefficients c_n and d_n are given by

$$c_n = -\frac{A_nV_n - {\rm i}C_nD_n}{W_nV_n + {\rm i}D_n^2}, \tag{S7}$$

$$d_n = -{\rm i}\frac{C_nW_n + A_nD_n}{W_nV_n + {\rm i}D_n^2}, \tag{S8}$$

where

$$A_n = {\rm i}\xi[\xi J_n'(\zeta)J_n(\xi) - \zeta J_n(\zeta)J_n'(\xi)], \tag{S9}$$

$$C_n = n\cos\theta\zeta J_n(\zeta)J_n(\xi)\left(\frac{\xi^2}{\zeta^2} - 1\right), \tag{S10}$$

$$D_n = n\cos\theta\zeta J_n(\zeta)H_n(\xi)\left(\frac{\xi^2}{\zeta^2} - 1\right), \tag{S11}$$

$$V_n = \varepsilon\xi^2 J_n'(\zeta)H_n(\xi) - \xi\zeta J_n(\zeta)H_n'(\xi), \tag{S12}$$

$$W_n = {\rm i}\xi\zeta J_n(\zeta)H_n'(\zeta) - {\rm i}\xi^2 J_n'(\zeta)H_n(\xi), \tag{S13}$$

and for which $\xi = k\rho_{\rm cyl}\sin\theta$ and $\zeta = k\rho_{\rm cyl}\sqrt{\varepsilon - \cos^2\theta}$. These expressions assume that the cylinder has vacuum permeability $\mu = 1$. The permittivity ε is in general a function of frequency ω. The scattered magnetic field can be calculated as

$$\mathbf{h}_{\rm sca}(\rho,\phi,z) = \frac{-{\rm i}}{c\mu_0}\sum_{n=-\infty}^{\infty} \frac{(-{\rm i})^n}{k\sin\theta}\left[{\rm i}c_n\mathbf{N}_n(\rho,\phi,z) + d_n\mathbf{M}_n(\rho,\phi,z)\right]. \tag{S14}$$

1.3 Internal field

The electric field internal to the cylinder takes the form

$$\mathbf{e}_{\rm int}(\rho,\phi,z) = \sum_{n=-\infty}^{\infty} \frac{(-{\rm i})^n}{k\sin\theta}[g_n\mathbf{M}_n(\rho,\phi,z) + f_n\mathbf{N}_n(\rho,\phi,z)] \tag{S15}$$

in which case the function $Z_n(a)$ generating $\mathbf{M}_n$ and $\mathbf{N}_n$ is $J_n(a)$ with argument $a = k\rho\sqrt{\varepsilon - \cos^2\theta}$. The coefficients g_n and f_n may be found by ensuring continuity of the internal electric field $\hat{\boldsymbol{\phi}}$ component with that of $\mathbf{e}_{\rm sca} + \mathbf{e}_{\rm inc}$ across the cylinder boundary:

$$f_n = d_n\frac{H_n(\xi)}{J_n(\zeta)}, \tag{S16}$$

$$g_n = \frac{1}{J_n'(\zeta)}\left[-n\cos\theta\left(d_n\frac{H_n(\xi)}{\xi} - f_n\frac{J_n(\zeta)}{\zeta}\right) + {\rm i}c_nH_n'(\xi) - {\rm i}J_n'(\xi)\right]. \tag{S17}$$

The internal magnetic field is

$$\mathbf{h}_{\rm int}(\rho,\phi,z) = \frac{-{\rm i}\sqrt{\varepsilon}}{c\mu_0}\sum_{n=-\infty}^{\infty} \frac{(-{\rm i})^n}{k\sin\theta}[g_n\mathbf{N}_n(\rho,\phi,z) + f_n\mathbf{M}_n(\rho,\phi,z)]. \tag{S18}$$

2 Trapping field configuration

We simulated the interaction between the infinite cylinder and two TE counter-propagating pulses, polarised in the xy plane. In this simulation, we consider the pulses normally incident, $\theta = \pi/2$, therefore, the fields are uniform along z. The cylinder permittivity is taken to be $\varepsilon = \varepsilon_{\text{eff}}$ from Eq. (5) in the main text. In time-domain simulations, the permittivity needs to be considered as a function of frequency ω [i.e., $\varepsilon(\omega)$]. In our simulations, we choose a dispersionless permittivity ε_{eff}, suitable for narrow-band trapping pulses.

The electric and magnetic fields produced by a single pulse propagating along $-\hat{\mathbf{x}}$ were found by integrating the total field from a single unit amplitude plane wave, $\mathbf{e}_{\text{tot}}$ and $\mathbf{h}_{\text{tot}}$, over two variables, angular frequency ω and the azimuthal angle of incidence φ according to a 2D Gaussian amplitude profile $A(\omega, \hat{k}_y)$, where $\hat{k}_y = -\sin\varphi$ for normal incidence ($\theta = \pi/2$). The electric field can be found as

$$\mathbf{E}_{\text{tot}}^{(\text{pulse})} = \int_{-\infty}^{\infty} \int_{-\pi/2}^{\pi/2} A(\omega, \sin\varphi)\mathbf{e}_{\text{tot}}(\omega, \varphi)\mathrm{e}^{\mathrm{i}\psi(\varphi)} d\varphi d\omega \,. \tag{S19}$$

The magnetic field has a similar expression. Here we have neglected notations of the co-ordinate dependence of $\mathbf{E}_{\text{tot}}^{(\text{pulse})}$ and $\mathbf{e}_{\text{tot}}$ and have instead emphasised the fact that, by way of the expressions in the preceding sections, $\mathbf{e}_{\text{tot}}$ depends both on ω and φ. The phase angle $\psi(\varphi) = -kx_0\cos\varphi - ky_0\sin\varphi$ enables the focal point of the pulse to be translated to a position (x_0, y_0) relative to the centre of the cylinder at the origin (this was the approach used to calculate optical force at various positions in a pulse spatial envelope, and subsequently in the envelope of the counter-propagating pulses). The amplitude function $A(\omega, \sin\varphi) = A_0 B(\omega) C(\sin\varphi)$ is composed of a normalisation factor A_0, controlling the absolute amplitude of the pulse, and two Gaussian functions, the first given by

$$B(\omega) = \frac{T}{\sqrt{2\pi}} \exp\left(-\frac{(\omega - \omega_c)^2 T^2}{4}\right), \tag{S20}$$

where T is the time interval between the pulse maximum energy and its maximum energy divided by e^2, which can be converted to mean the amplitude full width at half maximum (FWHM) by changing $T \to T\sqrt{-2/\ln 0.25}/2$. The second Gaussian function is

$$C(\sin\varphi) = \frac{w}{\sqrt{2\pi}} \exp\left(-\frac{(2\pi \sin\varphi)^2 w^2}{4}\right), \tag{S21}$$

where w is the pulse waist normalised by wavelength.

The field of a pulse with the same polarisation but propagating in the $+\hat{\mathbf{x}}$ direction (i.e., a mirror reflection of Eq. (S19) across $x = 0$) can be obtained from Eq. (S19), adding π to the limits of integration of φ and multiplying the integrand by -1. This new field can be added to that of Eq. (S19) to describe the trapping configuration with two counter-propagating pulses.

References

[1] C. F. Bohren and D. R. Huffman, *Absorption and Scattering of Light by Small Particles.* Wiley, 4 1998.
